# Pennsylvania

**Published:** 1896-06

**Authors:** 


### CONTROL WORK.

*Pennsylvania.* Influenza of a very severe type is epizoötic at Sewickley and vicinity in the western part of the State.

The prevalence of tuberculosis in western Pennsylvania and the reported sale of condemned cows for food have aroused the health authorities to vigorous measures.

The veterinarians of the Schuylkill Valley, fully alive to the duties devolving upon them as an organized body, have issued a very strong circular to the various Boards of Health in their district, urging the appointment of meat- and milk-inspectors, and a strict surveillance of all markets, slaughter-houses, and dairies. Many interesting statistics are given in the circular.

The western part of Pennsylvania, including the cities of Allegheny and Pittsburg, is much exercised over the prevalence of rabies, glanders, and bovine tuberculosis. The services of Messrs. McNeil and Lacock, City Veterinarians of Pittsburg and Allegheny, have been retained by the State Veterinary Sanitary Board to direct the management of the outbreaks.

A large number of dairies in the Lehigh Valley adjacent to Wilkesbarre, Scranton, and other towns are being examined for tuberculosis under direction of the State Sanitary Board.

Wilkesbarre will have the opportunity of judging the beneficial results of a pure and wholesome milk-supply through the organization of the Hygienic Milk Co., which will furnish milk from dairies kept in the most approved sanitary conditions. The cattle, to insure freedom from tuberculosis, are examined at stated intervals.

Sewickley will have some thirty head of cattle to destroy on account of tuberculosis, out of some six hundred tested with tuberculin. Dr. H. S. Jackson, of Sewickley, and Dr. Robert Jennings, of Pittsburg, have been conducting the investigations. Fifteen of the Economy herd are also under condemnation, and have been ordered destroyed by one of the Trustees of the Harmony Society. The Sewickley Board of Health is considering an ordinance requiring an examination of all herds furnishing milk to that district. More active State measures are considered necessary by the people and the Board of Health of Sewickley.

The fifth victim of hydrophobia is reported at Hazleton, Pa., where an outbreak of rabies of considerable extent occurred some weeks ago. It is now suggested that a general dog-killing crusade be inaugurated, which surely would be a stroke of false policy.

